# The Effect of Plasma on Bacteria and Normal Cells in Infected Wound

**DOI:** 10.1155/2022/1838202

**Published:** 2022-11-30

**Authors:** Yiqian Li, Lanlan Nie, Shaohui Jin, Chen Sun, Xinpei Lu

**Affiliations:** ^1^State Key Laboratory of Advanced Electromagnetic Engineering and Technology, School of Electrical and Electronic Engineering, Huazhong University of Science and Technology, 430074 Wuhan, China; ^2^The GBA National Institute for Nanotechnology Innovation, China

## Abstract

Infected wound is one of the most common and serious problem in wound management. Cold atmospheric plasma (CAP) is considered to have a good effect in wound healing as a new type medicine. However, there is a key issue that has not been addressed in the treatment of infected wounds by plasma. Bacteria are always found in the deep region of the wound. When plasma is used to treat wounds, it also acts on normal tissue cells while decontaminating. What is the difference between the same dose of plasma acting on bacteria and normal cells? In this study, the most common bacteria (S. aureus, P. aeruginosa, and E. coli) in infected wound and two kinds of normal skin cells (human keratinocyte and human skin fibroblasts (HSF)) were selected to study the difference of the effects of the same dose of plasma on bacteria and cells. The results reveal that three kinds of 10^6^ CFU mL bacteria could be effectively inactivated by 5 order after plasma treatment 3 min, and P. aeruginosa was more sensitive to plasma (could be inactivated 5 order after 2 min treatment). The 10^4^ mL keratinocyte and HSF were treated with the same dose of plasma; keratinocyte can maintain over 90% of the activity and HSF cells can maintain over 70% of the activity. Moreover, the level of collagen I secreted by HSF increased. Therefore, cells can remain a high activity when a plasma dose capable of inactivating bacteria is applied to them.

## 1. Introduction

**Figure 1:**
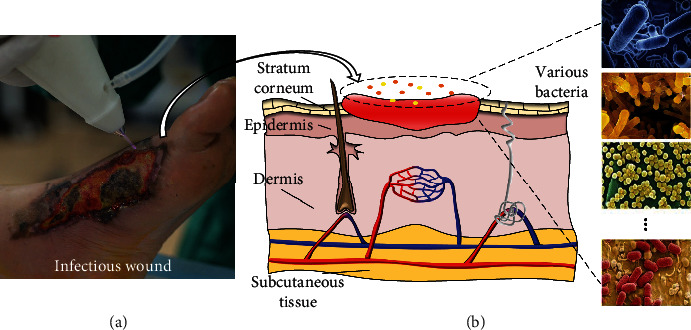
(a) The photo of infected wound. (b) The picture of skin structure.

In some developed countries, infected wounds are significant health issues. 1% of the population suffered from wounds continuously. And the cost of treatment for these wounds is about 2% to 3% of the total budget of the health care system [1, 2]. After injury, bacteria are always detected in the deep regions of the wound bed [3–5]. The attachment of bacteria to the wounds can be completed in just a few minutes [6]. Bacteria will damage every process of wound healing including coagulation, inflammation, proliferation, epithelialization, and remodeling. Higher bacterial level increases inflammation and delay the wound-healing process [3, 7, 8]. The pictures of infected wound and the skin structure are shown in Figure 1. Mupirocin antibiotic is widely used for the treatment of infectious wounds. However, the resistant of bacteria for mupirocin is increasing [5]. Now, there are many researches to find efficient and highly novel drugs to promote wound healing [5, 9–11]. Plasma as a new type of medicine has relatively clear biological effects including inactivation of a broad spectrum of bacteria, promoting cell proliferation, migration and differentiation at low doses, and promoting cell apoptosis at higher doses [6, 12].

Some studies have been carried out to learn the effects of plasma on wound healing. Isbary et al. [13] had used the plasma generator MicroPlaSter alpha and beta to treat patients with chronic wounds for 2 min a day. The devices could reduce the bacteria by 40% and 23.5% compared with the untreated group. Brehmer et al. [14] had used the PlasmaDerm VU-2010 device to treat 7 patients and another 7 patients received conventional treatment. Both groups received treatment 3 times a week for 8 weeks. 5 patients of the experimental group reduced wounds size. Among the 7 patients treated with plasma, bacteria load had been reduced significantly and 4 patients had a reduction in ulcer size. Plasma treatment has advantage in reducing wounds size and pain sensation.

In summary, one of the key reasons why plasma can promote infected wound is its killing effect on bacteria. However, another key question has not been addressed by researchers; that is, how the plasma impacts the normal cells while decontaminate. Some people has mentioned the concept of plasma doses on bacteria and cells [15, 16], but they did not treat them under the same conditions (including the power, processing method, processing area, and processing time).

In order to answer the above questions, we designed an in vitro experiment by treating common wound infection bacteria and normal skin tissue cells with plasma under the same conditions, then to detect the effect of plasma on it. Many studies have proved that S. aureus, P. aeruginosa, and E. coli are the main reasons for the delay of acute and chronic wound healing and infection [10, 17]. Fibroblasts and keratinocytes play an important role in the process of wound healing [14]. In the early stages of skin damage, keratinocytes begin to migrate and differentiate to the wound and produce a large number of cytokines, which can stimulate not only keratinocytes but also other inflammatory cells and fibroblasts to promote wound healing [18, 19]. Fibroblasts are connective tissue cells, which can generate collagen that can repair the defect and restore the anatomical structure to promote wound healing [14, 20]. It mainly secretes collagen I (85-90%) and collagen III (10-15%) [21].

In our experiment, we apply the plasma to a specific area, where bacteria and cells fill the space (a well with a diameter of 3.5 cm in the 6-well plates). It is important to emphasize that as the size of the HSF and keratinocyte is about ten microns, while the size of bacteria is about one micron, the number of bacteria should be set to 100 times the number of cells. And the liquid environment where cells and bacteria located during plasma treatment has been changed to PBS solution to eliminate the influence of different culture media. PBS is commonly used to simulate the in vivo environment when tested in vitro. The device we used is a new array type air plasma device which is touchable, safe, and has a large discharge area. The effects of plasma on bacteria and viability of cells would be measured through CFU-counting method and flow cytometry. The secretion of collagen I by fibroblasts was also measured. Through our work, we can conclude that bacteria can be inactivated while the cells still maintain a high activity under the same plasma power. And the ability of each fibroblast to secrete collagen I has been enhanced.

## 2. Materials and Methods

### 2.1. The DC-Driven CAP Array Source

**Figure 2:**
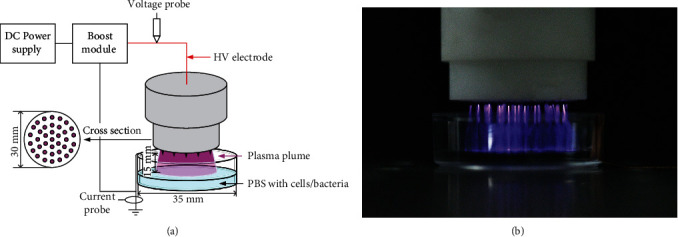
(a) Schematic of the discharge system to bacteria and cells. (b) The photo of the discharge array.

The device that generates plasma plume (Figure 2) is driven by a DC low-voltage source. The low voltage is raised to the kV level through a 1000-fold set up voltage transformer. 37 groups of high-voltage electrodes are connected thereafter. Each group of electrodes is welded by a 300 M*Ω* current-limiting resistor and a tungsten needle, and plasma is generated at the tip of the tungsten needle. The jet cross-section is about 700 mm^2^ which greatly increased the treatment area. The device is portable because it can discharge in the air. More importantly, there is no electric shock or obvious heat sensation in direct contact with the human body for several minutes.

**Figure 3:**
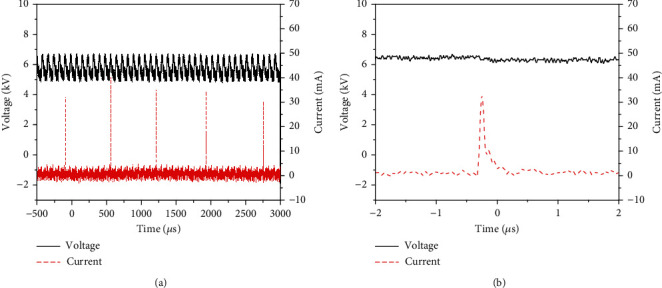
(a) Typical current-voltage waveforms of the plasma. (b) Zoomed view of a single pulse.

The distance between the tip of the tungsten needle that generated plasma and the liquid surface to be processed is fixed at 15 mm. The discharge current is the current flowing through the grounded wire measured by a current probe (Tektronix TCP312A) and the voltage is measured by a voltage probe (Tektronix P6015A). The waveforms of current and voltage are shown in Figure 3.  Figure 3(a) shows that the discharge appears as a periodic microsecond repetitive pulse and its repetition frequency is about 1.4 kHz. The applied voltage is about 5.74 kV. The peak current can reach 40 mA.  Figure 3(b) is a zoomed view of a typical single pulse.

The plasma power can be calculated through the bellow formula:

(1) $$\begin{array}{c} P_{\text{dis}}=f\int_{0}^{\tau }V\left(t\right)∙I\left(t\right)dt \end{array}$$

It can be calculated that the average power is about 1.16 W.

### 2.2. Chemical Analysis Methods of Aqueous Species

We chose to use a Hydrogen Peroxide Assay Kit (Beyotime, China) to detect the concentration of hydrogen peroxide. 50 *μ*L of the plasma-treated solution was taken and mixed with 100 *μ*L of hydrogen peroxide detection reagent. After 30 min of reaction, a microplate reader (BioTek, Synergy H1) was used to measure the absorbance at a wavelength of 560 nm. Finally, the concentration of hydrogen peroxide can be calculated according to the standard curve.

The concentration of nitrite and nitrate was detected through Nitrite Assay Kit (Nanjing Jiancheng, China) and Nitric Oxide Assay Kit (nitrate reductase method) (Nanjing Jiancheng, China), and their absorbance at a wavelength of 550 nm was detected by an ultraviolet spectrophotometer (UV1800PC, wavelength range of 190~1100 nm).

### 2.3. Reagents and Bacteria/Cells Culture

S. aureus (CMCC (B) 26003), E. coli (ATCC25922), and P. aeruginosa (CMCC (B) 10104) were provided by the College of Life Science and Technology, HUST. These bacteria were, respectively, seeded into Trypticase Soy Broth (TSB) media, in which S. aureus and E. coli were stored at 4°C, while P. aeruginosa was stored at room temperature.

The HSF and keratinocyte were provided by the College of Life Science and Technology, HUST. Both cells were cultured in Dulbecco's modified Eagle medium, high glucose (HyClone, America) containing 15% fetal bovine serum (Gibco, America) and 1% penicillin/streptomycin (Gibco, America). They were seeded in 6-well plates (Corning) with 2 mL in one well and cultured in a cell incubator (37°C, 5% CO_2_). To ensure cell viability, all experiments should be done when cells grew to fulfill 80~90% of the bottom of the 6-well plates within passages 3-6, and the culture media should be changed every 2 days.

### 2.4. Plasma Treatment of Bacteria/Cells

It is widely known that there is a large difference between the size of bacteria and cells. The size of the HSF and keratinocyte is about ten microns, while the size of bacteria is about one micron. When the same number of cells and bacteria is spread on the bottom of 6-well plates, the treated area varies greatly. Cells can fulfill the bottom, while bacteria can cover only 1% of the bottom. This obviously does not meet our requirements for processing cells and bacteria under the same conditions. To ensure consistency, the number of bacteria should be set to 100 times the number of cells. After consulting the literature and experiments, we know that it is appropriate when the number of cells seeded in each well of 6-well plates with 2 mL at a density of 1 × 10^4^ mL, so the number of bacteria should be set at 2 mL with a density of 1 × 10^6^ mL.

PBS was used instead of culture media (cells were cultured in DMEM, bacteria were cultured in TSA medium) during plasma treatment to eliminate the effects of different culture media. This process should be careful to reduce the loss of bacteria and cells. For bacteria, we used 2 mL of bacterial solution with a density of 1 × 10^6^ mL, centrifuged it at 10000 rpm for 10 min, discarded the supernatant carefully, then took 2 mL of PBS solution to resuspend the bacterial sediment and seeded it into one well of 6-well plates. For cells, since the cells are adherent cells, we could replace the old culture media with 2 mL PBS softly in each well with losing very little cells.

The 6-well plate which was added by bacteria solution or cells was placed on a grounded clean bench. And a wire was used to connect the solution with the ground. After plasma treatment, for bacteria, the treated solution was collected. 100 *μ*L of treated solution was plated on the Tryptose Soya Agar (TSA) media and placed it in a bacteria incubator (37°C) for about 24 h for subsequent CFU counting. For cells, some adherent cells may become no longer adherent and suspended in the PBS after plasma treatment. In order to reduce errors, we need to collect the treated PBS, centrifuged it at 1000 rpm for 5 min, discarded the supernatant, and used 1 mL of fresh culture media to resuspend the cell pellet. Then 1 mL of cells resuspension and another 1 mL of fresh culture media were added to one well of the 6-well plate with the treated cells. Finally, cells were put back into the cell incubator and cultivated for 24 h and 48 h.

### 2.5. Cell Viability Assay

The Annexin V-FITC Apoptosis Detection Kit (Dojindo Laboratories, Japan) for CytoFLEX (Beckman Coulter, America) was used to distinguish viable cells, apoptotic cells, and necrosis cells. The specific process was as follows.

After the plasma-treated cells had been cultured for 24 h and 48 h, respectively, these cells (including the cell supernatant) were digested with trypsin (without EDTA) (Gibco, America) and collected. We had added 3 mL of PBS and centrifuged it at 1000 rpm for 3 min, then discarded the supernatant. The cell pellet was resuspended with 300 *μ*L of binding buffer. 5 *μ*L of Annexin V-FITC and 5 *μ*L of PI solution were added to the cell suspension. And it was incubated for 15 min in the dark at room temperature. Finally, the results should be tested within 1 h.

Trypan blue is a regent which can differentiate the cell viability. The cells which can be stained to blue were dead. We use the Trypan Blue Staining Cell Viability Assay Kit (Beyotime, China) to calculate the number of viable cells after plasma treatment.

### 2.6. The Collagen I Secretion of HSF

The supernatants of HSF after plasma treatment for 24 and 48 h were collected, and the concentration of collagen I was detected using a microplate reader at 450 nm wavelength with Human Collagen Type I Elisa Kit (Nanjing Jiancheng, China).

### 2.7. Statistical Analysis

Each experiment sets up three parallel experiments. All data were processed with SPSS computer programs (version 21.0 for Windows) by one-way analysis of variance (ANOVA). The results are expressed as the mean ± standard deviation. *P* < 0.05 was considered statistically significant, while *P* < 0.01 was extremely significant.

## 3. Results

### 3.1. The Inactivation Effect of Plasma on Bacteria

**Figure 4:**
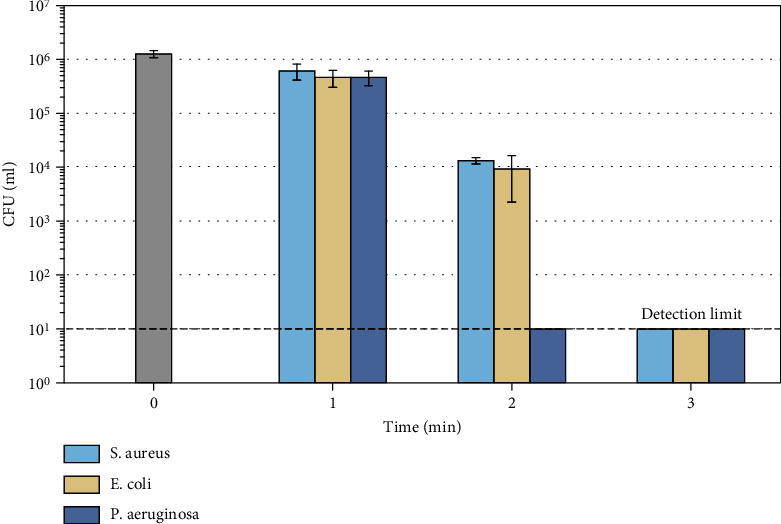
Results of CAP treatment of bacteria.

We choose to use the CFU-counting method to quantify the inactivation efficiency of plasma on bacteria, the results were shown in Figure 4. The initial concentrations of bacteria were all 10^6^ mL. With the increase of CAP treatment time, the concentration of the 3 kinds of bacteria showed a downward trend. While with one minute of plasma treatment, the decrease of the 3 kinds of bacteria was very small, less than 1 log. When the treatment time reached 2 min, there was a sudden decrease in the amount of these bacteria. E. coli and S. aureus could reduce almost 2 logs and P. aeruginosa could be all inactivated, while it took 3 min for plasma to inactivate 5 logs of E. coli and S. aureus. Through the results, we could find that plasma has a greater inactivation effect on P. aeruginosa than E. coli and S. aureus.

### 3.2. The Effect of Plasma on Cell Viability and Collagen Secretion

**Figure 5:**
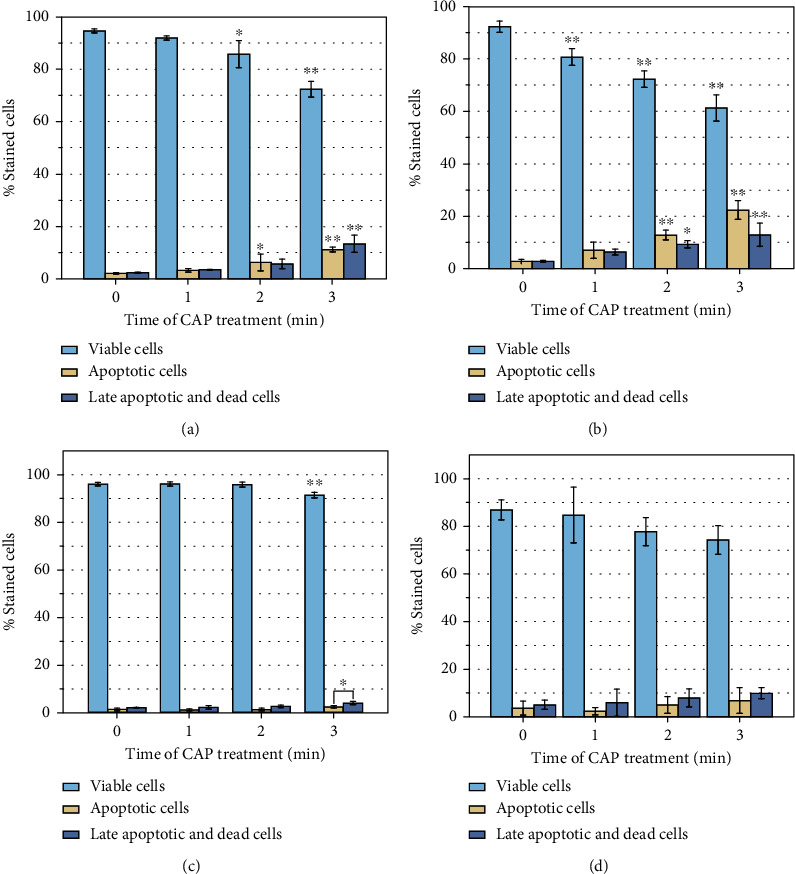
Results of CAP treatment of HSF and keratinocyte: 0, 1, 2, and 3 min, counted 24 and 48 h posttreatment, where *n* = 3, ^∗^*P* < 0.05, ^∗∗^*P* < 0.01. (a) HSF activity after plasma treatment for 24 h. (b) HSF activity after plasma treatment for 48 h. (c) Keratinocyte activity after plasma treatment for 24 h. (d) Keratinocyte activity after plasma treatment for 48 h.

**Figure 6:**
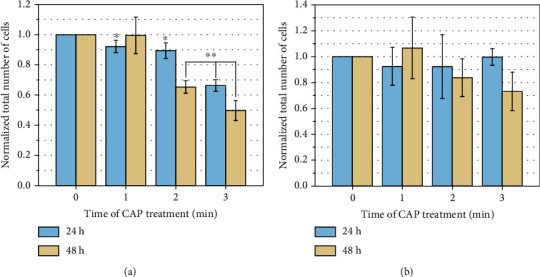
Results of CAP treatment of HSF and keratinocyte: 0, 1, 2, and 3 min, counted 24 and 48 h post treatment, where *n* = 3, ^∗^*P* < 0.05, ^∗∗^*P* < 0.01. (a) HSF. (b) Keratinocyte.

The results of cell viability of HSF and keratinocyte after CAP treatment are shown in Figures 5 and 6.

In Figure 5, for HSF, with the increase of the treatment time, the percentage of viable cells continues to decrease. However, the percentage of apoptotic cells, late apoptotic, and necrosis cells are constantly rising. The percentage of apoptotic cells is lower than the percentage of late apoptotic and necrosis cells after 24 h of incubation, while the situation is opposite after 48 h. This suggests that increasing plasma treatment time may stimulate the expression of apoptotic programs. So the excessive plasma treatment should be avoided.

For keratinocyte, whether they were cultured for 24 h or 48 h after plasma treatment, the cells can maintain good activities. While after 48 hours of culture, the number of viable cells decreased compared to 24 hours of culture. And the percentage of apoptotic cells is lower than the percentage of late apoptotic and necrosis cells. From these data, we can find that keratinocyte is more tolerant to cold atmospheric plasma treatment than HSF. After plasma treatment and culture for 24 hours, the proportion of viable cells can still be maintained at 90%.

Figure 5 can only explain the percentage of viable cells in the total cells. It may happen that after treatment, although the proportion of viable cells has decreased, while their number has increased compared with control group. In order to clarify this, the number of viable cells was counted with trypan blue. The number of viable cells in the control group was normalized to 1 for easy comparison. The results are shown in Figure 6.

In Figure 6, for HSF, whatever the incubation time is 24 h or 48 h, as the CAP treatment time increases, the number of viable cells continues to decrease. When we treated it for 1 min and incubated for 24 or 48 h, or treated it for 2 min with a 24 h incubation, the number of HSF does not show an obvious decrease. While when we treated it for 2 min and incubated for 48 h, or treated it for 3 min with 24 or 48 h of incubation, the number of HSF show a dramatic decrease. Especially when the cells were treated for 3 min and then cultured for 48 h, the number of viable cells was decreased to only 50% compared with the control group, which indicates that 3 min of plasma treatment may be too long for HSF.

For keratinocyte, there is no significant difference between the number of viable cells in the experimental group and the control group (*P* > 0.05), which can prove that the keratinocyte is not that sensitive to plasma compared with HSF.

We can find that when plasma treatment time under 2 min will not decrease the number of the viable cells after 48 h. While the longer plasma treatment time will cause great damage to cells after 48 h of incubation than 24 h.

**Figure 7:**
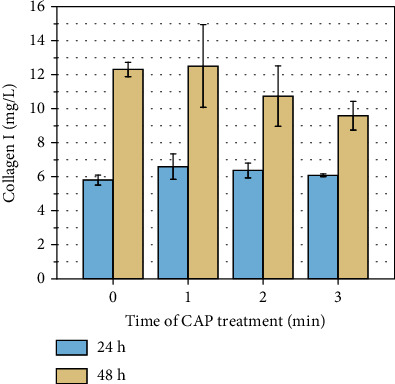
The secretion of collagen I after CAP treatment, where *n* = 3, ^∗^*P* < 0.05, ^∗∗^*P* < 0.01.

Collagen plays an important role in promoting wound healing. We chose to detect the secretion of collagen I by HSF after plasma treatment. The result of the amount of collagen I secreted by HSF is shown in Figure 7. There is no significant difference in collagen I secretion after CAP treatment compared with the control group (*P* > 0.05). After plasma treatment for 3 min and incubated 24 h, the number of cells was 66% of the control group as shown in Figure 6, but the amount of collagen I secretion did not change a lot which indicated that the ability of each cell to secrete collagen I was enhanced greatly under plasma stimulation. After plasma treatment for 3 min and incubated 48 h, the secretion of collagen I was reduced to 80% of the control group, the number of viable cells was 50% of the control group, so the ability of each cell to secrete collagen I was also enhanced.

### 3.3. Reactive Species in Aqueous Phase

**Figure 8:**
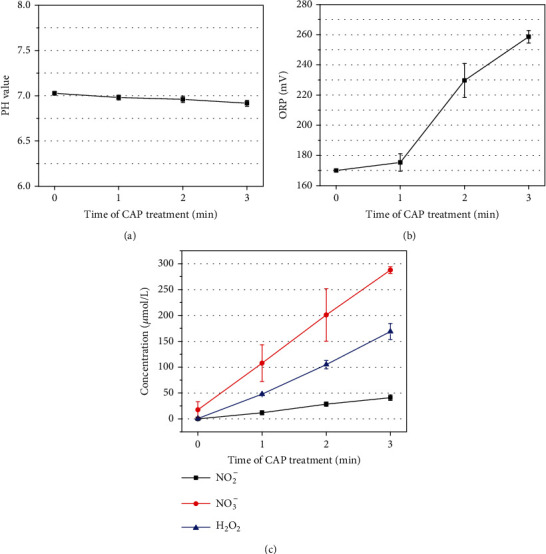
(a) pH. (b) ORP. (c) Long-lived reactive species.

The pH, Oxidation-Reduction Potential (ORP), and reactive species in aqueous phase have been measured as shown in Figure 8, which reflect the long lifetime active components after plasma treatment. In the aqueous phase, due to the ability of PBS solution to buffer pH, the pH basically does not change and is maintained at about 7 when the PBS solution is treated by plasma. The ORP increases with the increase of CAP treatment time, and the maximum ORP is 259 mV. The ORP value basically has no bactericidal ability when the pH is 7. During the plasma treatment, the concentration of nitrite, nitrate, and hydrogen peroxide has accumulated continuously and reached 40.9 ± 5.33 *μ*m, 287.75 ± 6.26 *μ*m, and 168.94 ± 15.51 *μ*m at 3 min.

## 4. Discussion

The use of plasma in the treatment of infected wounds has received increasing attention [22–26]. As an efficient medicine, its biological mechanism at the cellular level has been studied by some researchers [6, 12, 20, 27]. Existing studies have shown that plasma has a strong antibacterial ability, which can reduce the bacterial load at the wound [13, 14], promote the proliferation and migration of cells by stimulating the secretion of cytokines, and promote the production of blood vessels [28]. But the conclusion is not obtained under the same plasma parameters, and they did not control the treated area and liquid environment of cells and bacteria to be equal. Plasma doses that kill bacteria may do a lot of damage to cells, and plasma doses that promote cell proliferation may not kill bacteria. People want to know whether normal cells can be harmed while decontaminating.

Through our work, an air array plasma device has been used to treat cells and bacteria under the same environment and the same electrical parameters. The number of treated cells and bacteria and the experimental process have been decided to ensure the consistency of treated area. Under the same electrical parameters and the same area treated, the selected three bacteria can be completely decontaminated within 3 minutes, but HSF and keratinocyte can still maintain high activity. In our experiments, there were also differences between bacteria and cells when treating by plasma. S. aureus and E. coli took plasma 3 minutes to kill, while P. aeruginosa took only 2 minutes. The keratinocyte can maintain about 90% of the activity after plasma treatment for 3 minutes, while the HSF cells can only maintain 70% of the activity, but the total amount of collagen secreted remains basically unchanged, and the ability of a single HSF cell to secrete collagen hugely improvements. Through the results, we can also find that excessive plasma treatment will cause damage to cells with a longer time of incubation.

The efficacy of CAP in the biological applications might rely on the combined action of the reactive oxygen species (ROS), reactive nitrogen species (RNS), free radicals, UV photons, charged particles, and electric fields [29–31]. Plasma can produce a large number of reactive oxygen and nitrogen species (RONS) during the discharge process, among which ROS mainly includes OH∙, O_2_^.−^, O_3_, O_2_(1Δ_g_), and H_2_O_2_ and RNS mainly includes ONOO^−^, NO_2_^−^, and NO_3_^−^. The effect of the electric field is also an important factor when the plasma treats directly. These articles [32–35] introduce the experimental measurement and simulation of the transient electric field characteristics in the study of cold atmospheric plasma. UV radiation also contributes during plasma treatment, which is thought to cause dimerization of thymine bases in DNA strands, which inhibits the bacteria's ability to replicate properly [36].

Oxidative stress may be the main reason leading to cell death [37]. These reactive species generated by plasma may modify or destroy the integrity of cell membranes by physical or chemical ways. Lipids are the most easily oxidized macromolecules in cell membranes, and lipid peroxidation (LPO) of membranes can cause cytosolic leakage. LPO is a chain reaction that produces MDA (malondialdehyde). MDA is considered as an indicator to characterize the effectiveness of LPO. Plasma treatment results in a significant increase in MDA levels in cells [37, 38]. Joshi observed a reduction in the morphology of E. coli by treating E. coli with plasma, a typical behavior by reducing surface area to minimize metabolic activity and energy requirements, which are often observed when cells are subjected to severe oxidative stress. Yan et al. [39] used plasma plume to treat HepG2 cells. It has been proposed that intracellular reactive oxygen species would attack the unsaturated fatty acid side chains of membrane lipids, leading to the formation of lipid hydroperoxides. The accumulation of lipid hydroperoxides in the cell membrane disrupts the normal function of the cell and leads to the rupture of the cell membrane, which eventually leads to severe cytoplasmic leakage [40]. And plasma treatment can cause cell cycle arrest in G2/M phase [39].

In this experiment, the cells can still maintain good activity under the plasma parameters that can kill bacteria. We speculate the reasons of the different tolerance between cells and bacteria to plasma as follows. Cells have a more advanced adaptive mechanism than bacteria. Studies have pointed out that the electrostatic pressure in the bacteria accumulated after plasma treatment will destroy the bacterial wall structure, while the cells have more strong functions which can adapt to local changes in the electrical environment caused by plasma [15, 41]. Cells have a more complicated defense mechanism against superoxide which play an important role in the peroxidation of phospholipid bilayer which will lead to DNA damaged [39]. Cells can convert superoxide into less harmful hydrogen peroxide and repair the DNA damage, while bacteria cannot do so due to lack of enough related enzyme [42]. And study has proved that the concentration of hydrogen peroxide that can cause DNA double strand breaks is equal to 10 *μ*m [28]. We have measured the concentration of hydrogen peroxide which was shown in Figure 8. The concentration of hydrogen peroxide is 0.34 *μ*m which will cause no harm to cells. Cells have a lower surface to volume ratio than bacteria which mean that the surface of cells will suffer less damage from UV and electric field. And more importantly, the lower surface to volume ratio means more adaptive to oxidative stress [43]. Bacteria always replicate frequently and have no nucleus, so its DNA is exposed which would be damaged and unfolded easily under the plasma treatment [16]. It is not just cells and bacteria that differ in their tolerance to plasma. There is a different inactivation time between different bacteria. The gram-positive bacteria is more difficult to kill based on the present research [41]. Gram-positive cell wall (20–80 nm) is thicker and with a higher rigidity than the gram-negative cell wall (1.5-10 nm). The gram-negative cell surfaces are rough and irregular due to the presence of an outer membrane so that it could be more sensitive to electrostatic disruption [15, 44].

Numerous studies have shown that nitric oxide is an important molecule in promoting wound healing [45–47]. Nitric oxide can promote wound healing by promoting HSF migration and collagen secretion [47]. The concentration level of nitric oxide in the aqueous phase should be represented by the sum of nitrite and nitrate. In our experiment, the concentration of nitric oxide(NO_2_^−^ + NO_3_^−^) in plasma-activated PBS is about 328 *μ*m.

Plasma treatment for a longer time (3 min) will cause greater damage to the cells within 48 h incubation, which indicated that the excessive plasma will cause sustained damage to cells and it may influence the progeny cells through accumulated genomic instability [48, 49]. So, it is very important to find a suitable plasma treatment time which can be safely used. Our experiment proved that bacteria are more sensitive to plasma than cells under the same conditions. While excessively plasma treatment should be avoided to prevent damage to cells, in clinical application, the situation is very complicated and the in vitro protocol cannot be directly applied. The impedance factor of the human body should also be considered [50]. Therefore, this topic can be further explored.

Cold atmospheric plasma has the advantage of broad-spectrum killing of germs as a new type of infected wound treatment and can be indiscriminately treated for different bacteria. Rapid, convenient, and highly effective treatment can be achieved using plasma therapy. In addition, the mechanism of plasma killing bacteria is based on a combination of chemical and physical effects [51], so its mechanism is very complicated. More importantly, after plasma treatment, cells can still maintain good activity and collagen secretion capacity.

## 5. Conclusion

Plasma, as an efficient type of medicine, is widely used in wound healing. Experiments have verified that the same plasma doses could inactivate bacteria while the cells still maintain high activities. And each cell can secrete more collagen I. All in all, the difference between biological effects of plasma on cells and bacteria is very important to the study of infected wound-healing mechanisms, and it can provide a reference for determining the best plasma treatment time.

## Data Availability

All data supporting the results are provided in the text and figures and are available from the author.
